# P-766. Evaluating clinical outcomes of susceptible vs non-susceptible empiric antibiotic therapy for extended-spectrum β-lactamase producing Enterobacterales urinary tract infections - a case for optimizing antimicrobial stewardship

**DOI:** 10.1093/ofid/ofaf695.977

**Published:** 2026-01-11

**Authors:** David T Adams, Satwinder S Kaur, Casey L Schrank

**Affiliations:** Texas Health Harris Methodist Hospital Fort Worth, Fort Worth, TX; Texas Health Harris Methodist Hospital Fort Worth, Fort Worth, TX; Texas Health Harris Methodist Fort Worth, Burleson, Texas

## Abstract

**Background:**

Data comparing clinical outcomes is limited in patients receiving empiric ceftriaxone versus carbapenems for confirmed ESBL UTIs. Given the prevalence and rising resistance rates of *Enterobacterales spp.* causing UTIs, empiric carbapenem use becomes increasingly utilized. While carbapenems remain the current standard of definitive care for ESBL infections, it is unclear whether their empiric use leads to improved clinical outcomes in patients with UTIs compared to empiric regimens lacking activity against ESBL isolates.

**Methods:**

A retrospective cohort study conducted at Texas Health Harris Methodist Hospital Fort Worth in patients with confirmed ESBL UTIs grouped by whether empiric therapy was determined to be susceptible (EMP-S) or nonsusceptible (EMP-NS) to the isolate per the final susceptibility report. Patients with concurrent sources of infection, pregnant, or received empiric cefepime or piperacillin/tazobactam were excluded. The primary clinical outcome evaluated was a composite of worsening symptoms documented by a provider and hemodynamic stability on the day that the ESBL isolate was identified. Other outcomes included in-hospital mortality and 30-day readmission for a UTI.

**Results:**

214 patients met inclusion (124 and 90 in the EMP-NS and EMP-S groups, respectively) with ceftriaxone used most in the EMP-NS group and carbapenems in the EMP-S group. No significant difference was observed in patients meeting criteria for the primary outcome between the EMP-NS and EMP-S groups (25.0% vs 32.2%, p = 0.246). A higher baseline rate of sepsis was observed in the EMP-S group. When comparing the 51 patients in each group that had sepsis, there was no difference observed in the primary outcome between the EMP-NS and EMP-S groups (47.1% vs 54.9%, p = 0.428). No significant differences were observed regarding in-hospital mortality and 30-day readmissions due to a UTI between groups.

**Conclusion:**

Empiric antibiotic therapy later confirmed to be nonsusceptible showed no difference in clinical outcomes compared to empiric carbapenems for ESBL UTIs. Further evaluation of this approach could help offer a practical framework to preserve carbapenem efficacy through culture-guided therapy rather than empiric broad-spectrum use for a frequently encountered infectious etiology.

**Disclosures:**

All Authors: No reported disclosures

